# Controlling congenital and paediatric chagas disease through a community health approach with active surveillance and promotion of paediatric awareness

**DOI:** 10.1186/1471-2458-14-1201

**Published:** 2014-11-21

**Authors:** Antoni Soriano-Arandes, Luca Basile, Hakima Ouaarab, Isabel Clavería, Jordi Gómez i Prat, Juan Cabezos, Pilar Ciruela, Pedro Albajar-Viñas, Mireia Jané

**Affiliations:** Unitat de Salut Internacional. PROSICS, Programa Especial de Malalties Infeccioses Vall d’Hebron-Drassanes, Barcelona, Spain; Subdirecció General de Vigilància i Resposta a Emergències de Salut Pública, Agència de Salut Pública de Catalunya, Barcelona, Spain; Department of HIV/AIDS, Tuberculosis, Malaria and Neglected Diseases, Control of Neglected Tropical Diseases, WHO, Geneva, Switzerland

**Keywords:** Congenital chagas disease, Community health activity, *Trypanosoma cruzi*, Active surveillance

## Abstract

**Background:**

Chagas disease (CD) is endemic in countries of continental Latin America. Congenital transmission is a major concern worldwide. In 2010, the Public Health Agency of Catalonia (ASPCAT) launched a screening protocol for *Trypanosoma cruzi* infection in pregnant women and their newborns. In 2012, ASPCAT detected appropriate follow-up of pregnant women but incomplete information about their offspring.

**Methods:**

The PROSICS community health team carried out active surveillance and community health action in target populations. These activities included active case searches, group awareness workshops and visualization campaigns as well as investigation of all lost children born from pregnant women with CD and their families.

**Results:**

Overall, 42/179 (23.5%) cases were included in the study: 35/42 (83.3%) children were born in Hospitalet de Llobregat (Catalonia, Spain); 4/42 (16.7%) were born in Latin America; two were miscarried and one was stillborn. The mean age of pregnant women was 31.3 years (SD 5.52; range: 21–44): 90.5% were Bolivian, of whom 74% were diagnosed with CD during pregnancy. Of the 35 newborns, 31 were recovered by community health action; 12/31 were correctly controlled at Hospitalet de Llobregat and 19/31 were controlled at a primary health centre. Of these 19 (73.7%) cases, 14 were not tested for CD by family paediatricians and were recovered by the PROSICS community health team. Finally, two (6.9%) of the 29 newborns tested with serology were positive.

**Conclusions:**

It is essential to implement active surveillance, education and information activities at paediatric primary care and community levels to avoid the loss of CD-infected mothers and their newborns. Training sessions addressed to paediatricians and other involved health professionals would consolidate surveillance and care reference circuits, improving the control of congenital CD.

## Background

Chagas disease (CD) is a parasitic disease caused by *Trypanosoma cruzi*. Infection acquired through contact with the contaminated faeces of triatomine bugs has been the predominant route of transmission in Latin American continental countries [1]. Prevalence is estimated in around 8 million people and annual mortality in more than 10,000 people [2]. The distribution of the disease is heterogeneous along the American continent, being rural, periurban populations, and urban with low economic status in South American countries the most affected by CD [3, 4].

In most infected patients the disease evolves silently and chronically without any clinical symptoms. This characteristic, which differs from other neglected tropical diseases, is important for public health policies to prevent transmission and control the disease in affected countries. Of those infected with *T. cruzi*, however, 30–40% develop cardiac, intestinal, neurological or mixed chronic clinical manifestations [1], imposing an important cumulative morbidity and mortality on affected families and a heavy burden on the health systems of endemic and non-endemic countries.

Important epidemiological changes in the disease have occurred during the past decades [5, 6]. After a series of successful vectorial control achievements in many Latin American countries, other transmission routes – such as blood transfusion, congenital, oral, and organ transplantation – have become more prominent since the 1990s. Furthermore, the spread of the disease due to urbanization and increased population movements between Latin America and other continents has highlighted the importance of detection and prevention in non-endemic countries [7]. In response to this new epidemiological context, Spain launched Royal Decree 1008/2005 to screen *T. cruzi* infection in all blood donors arriving from endemic areas or in those previously subjected to blood transfusions in Latin American continental countries [8]. However, another transmission route – congenital transmission (i.e. transmission from infected mothers to their children) – is already an important challenge for Spain and other endemic and non-endemic countries [9]. A recent article aims to assess the frequency of congenital *T. cruzi* transmission [10].

The first case of congenital Chagas disease in Spain was detected in 2006 [11]. Since then, it has been estimated that the rate of congenital CD among people at risk of the infection is 0–13.8% in Spain [12]. Taking into account these rates and the fecundity rate for Latin American women in Europe, it is estimated that the number of pregnant women with CD in Spain would be of 1,400 per year and the number of *T. cruzi* infected newborns between 53 and 93 per year [13].

**Figure 1:**
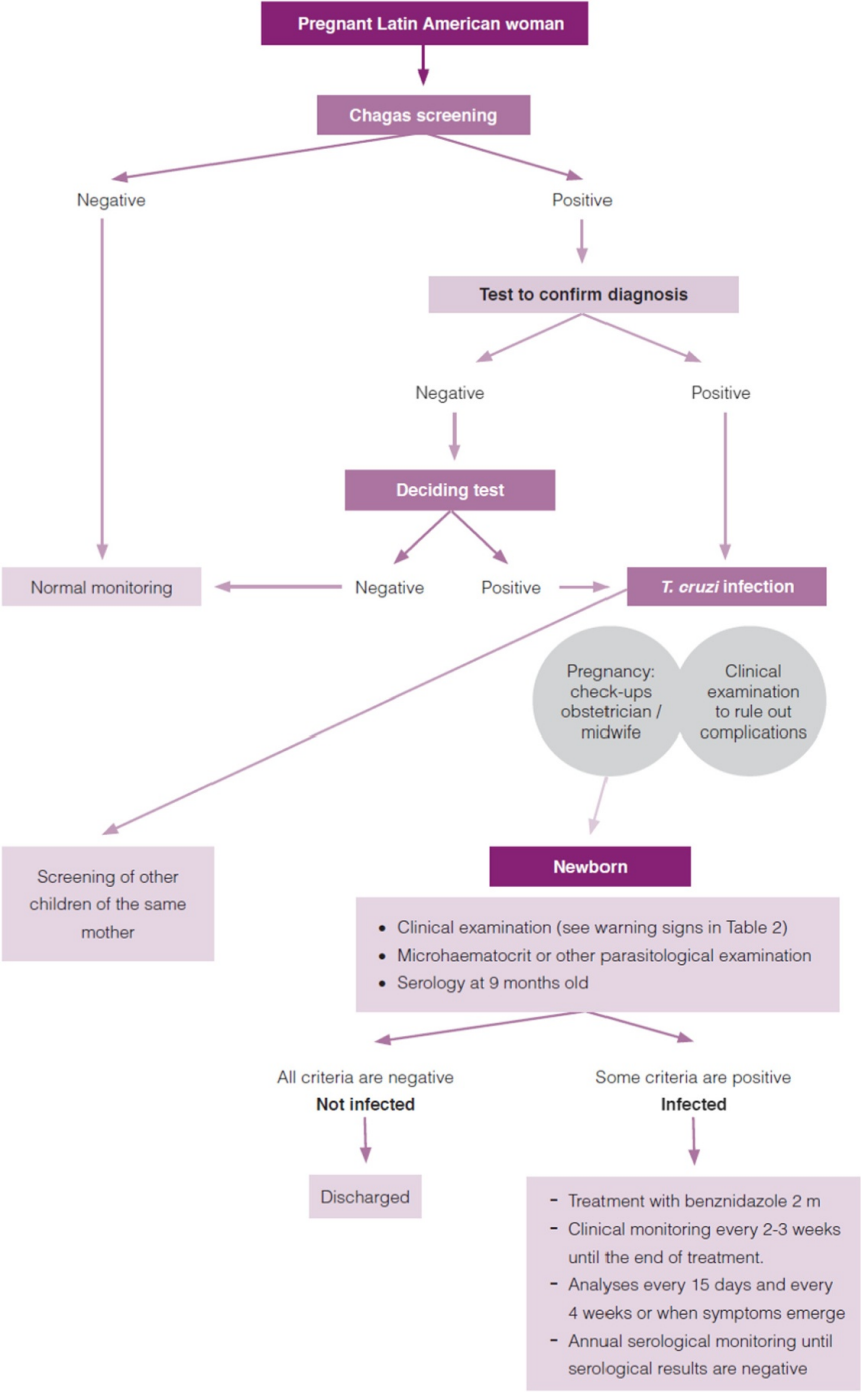
**Pathway for screening and diagnosis in pregnant women and newborns.** (Source: Protocol for screening and diagnosing Chagas disease in pregnant Latin American and their newborns.pdf; accessed 6 May 2014).

Given the potential risk of congenital CD in Catalonia, the Public Health Agency of Catalonia (ASPCAT) launched the “Protocol for screening and diagnosing Chagas disease in pregnant Latin American and their newborns” in 2010 [13], targeting Latin American pregnant women attending antenatal consultations in Catalonia (Figure 1). The aim of the Protocol is to detect *T. cruzi*-positive pregnant women and follow up their offspring until 9 months of age in order to elucidate their definitive CD status. The implementation of information, education and communication activities was planned, including training of health professionals working with CD detection and treatment, and specific sessions for those working in health centres with prenatal activities. Midwives, nurses, obstetricians, primary health care physicians and paediatricians attending these sessions were trained on *T. cruzi* infection, diagnosis and its care, with focus on congenital CD. According to the 2012 report of the Statistical Institute of Catalonia (IDESCAT), 333,703 Latin American immigrants were living in Catalonia (15.7% from Bolivia), 6,795 newborns (15.4% from Bolivia) were delivered by pregnant women at risk of CD and an estimated 203–387 pregnant women (90% from Bolivia) were infected with *T. cruzi*. The expected number of *T. cruzi*-infected newborns was therefore 7–16 per year [14].

In order to monitor the implementation of the Protocol, ASPCAT declared a total of 119 CD pregnant women who had given birth in Catalonia, and 20% (24/119) of the newborns were lost before serological control at 9 months of age in 2010 [15]. In 2011, 179 pregnant women were diagnosed with CD in Catalonia: 24% (43/179) came from one of the Catalan areas with the highest prevalence of the affected population, the Municipality of Hospitalet del Llobregat, with >250,000 inhabitants including 10% from endemic areas [16] without any CD paediatric referral.

Community-based and culturally-sensitive actions to manage CD in Europe are necessary because most people at risk are migrants who have frequent difficulties in accessing appropriate care. Furthermore, there are important challenges in screening newborns aged over 9 months after birth since the Protocol was launched in 2010 because diagnosis must be made with delayed serological tests. The aim of the study was to understand the reasons for lack of or deficient follow up of newborns from *T.cruzi*-infected mothers, and to apply and evaluate an active strategy to detect *T.cruzi* infection among those born from *T. cruzi* infected mothers. ASPCAT, in coordination with the Unit of Tropical Medicine and International Health of Vall Hebron-Drassanes, International Health Programme of the Catalan Institute of Health-PROSICS, planned a community health approach with active surveillance to know the final serological status and health condition of these children in relation to *T. cruzi* infection.

## Methods

Three meetings were held between ASPCAT and PROSICS before the activities were initiated. One of the inclusion criteria for active surveillance was being a *T. cruzi*-infected pregnant woman resident in Hospitalet de Llobregat and diagnosed before or during pregnancy. The first step of active surveillance was to know the final status of *T. cruzi* infection of children born from these women. If the children were not controlled, the PROSICS community health team solicited a visit with the family to test the newborns and inform the family about CD.

The presence of a PROSICS community health team consisting of a paediatrician, two community health nurses, a family physician working inside the community and community health workers (CHW) with 20 years of experience in the field of community mobilization, and especially in CD since 2006, helped to find agreement on the following two areas of work and provided a comprehensive response.

1. **Close active control of children born from CD-positive mothers**

When ASPCAT identified the list of *T.cruzi*-infected pregnant women resident in Hospitalet de Llobregat in 2011 with no information about birth and possible newborn follow-up, the PROSICS community health team was consulted about starting active surveillance. Some CHW activities were addressed to the Bolivian community. First, CHW tried to locate the family by telephoning at different times and days of the week (maximum 10 calls: morning, afternoon, evening and weekends). When this activity failed, either because the telephone number was not available or it was never answered, the CHW went to the home (maximum 2 visits) to find any relative of the CD case; finally, when the CHW found nobody at home, the neighbours were asked if anyone was living there and a note was left to call or to be addressed to PROSICS. If the CHW did not locate the mother via telephone or at home, PROSICS tried to find her in those community spaces frequented by the Latin American population.

The designated CHW for this activity belongs to the same Latin American community, facilitating knowledge of this particular environment, its needs, problems, fears, relationships, language and codes, and other forms of communication.

Once the CHW located the mother, he took information about the hospital of birth, parasitological controls at birth, serological control at 9 months and primary health care follow up. The PROSICS paediatrician coordinated with the community nurses, made an appointment for a follow-up visit and a *T. cruzi* serological test of uncontrolled children. Appointments were adapted to the mother’s schedule because incompatible work times are one of the reasons why mothers do not seek health control visits.

2. **Awareness and display of CD to the Latin American community**

PROSICS organized health promotion activities for Latin American mothers living in the city of Hospitalet del Llobregat. These included group workshops/sessions held in social spaces (associations, partner health centres, homes, community centres, community facilities) and community awareness campaigns (fairs, cultural events, recreational activities, newspapers for the target population) with audiovisual material about CD. During these activities the population was invited to the health centre for screening in order to detect and track CD cases.

The participation and involvement of various non-profit entities, partners, health services, religious leaders, government and other stakeholders in various health education activities was key to developing these community-based actions.

A database was built including the most important variables related to mothers (date of birth, age, family address, country of origin, telephone number, year of arrival in Spain, CD phase, primary health care centre, laboratory where CD diagnosis was obtained, and date of CD diagnosis), delivery of newborns (date, hospital, confirmation of the delivery, screening of the newborn at birth, and methods used for CD screening), and follow up of children (cause of loss to follow up obtained by the CHW and the paediatrician).

Ethical clearance for this research was not performed because data registration of the children born to a CD mother is mandatory following the 2010 launch in Catalonia of the Protocol.

## Results

Overall, 43/179 (24%) cases of *T.cruzi*-infected pregnant women who gave birth in 2011 met the inclusion criteria for the study. One case was excluded because negative CD status was later confirmed. Of these total cases, 35/42 (83.3%) pregnant women gave birth to a live newborn in Catalonia; 4/42 (16.7%) returned to their country of origin before delivery; 2/42 miscarried and 1/42 was stillborn. Regarding the country of origin, 90.5% (38/42) were Bolivian pregnant women. A total of 67% of pregnant women were in the chronic phase and indeterminate form. The mean age was 31.3 (SD 5.52; range: 21–44). At the time of CD diagnosis, 74% of cases were diagnosed during gestation, 21% were diagnosed before gestation, and none was diagnosed during delivery. The paediatrician of PROSICS recovered follow-up information from 31/35 identified newborns; the remaining 4 cases probably left Catalonia between birth and surveillance. Of the 31 recovered infants, 12 were regularly controlled at 9 months of age in hospitals outside the city of Hospitalet de Llobregat, but this information was not communicated to ASPCAT. The other 19 births in the General Hospital of Hospitalet de Llobregat, should have been controlled in primary health centres because there was no CD paediatric referent in that hospital. Only 5/19 were correctly followed up by family paediatricians; the remaining 14 (73.7%) were therefore not correctly tested for *T. cruzi* at 9 months of age. When parents were asked why their children had not been screened at a primary health centre, the response was that neither the paediatrician nor the paediatric nurses had been reminded to solicit *T. cruzi* serology at that age. As a result, 14 children with unknown serological status underwent serology control for *T. cruzi* by the PROSICS paediatrician but 2/14 did not attend the visit and were not tested for CD.

Finally, of the 29/31 newborns without information about serological status 2 were positive, which means a congenital CD transmission rate of 6.9%.

**Figure 2:**
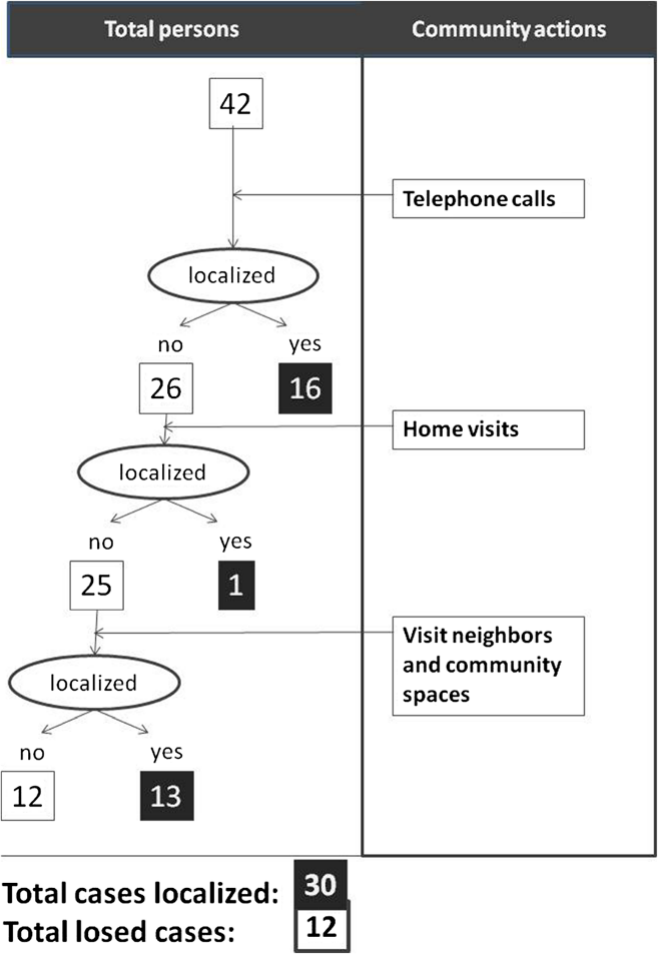
**Summary of congenital Chagas disease community health actions.**

Community health action recovered 73.8% (31/42) of the CD cases for whom final information had not been communicated to the ASPCAT through telephone calls (17 cases), community activities (13 cases) and home visits (1 case). The results of follow-up and community health action are shown in Table 1 and Figure 2 respectively.

**Table 1:** **Summary of follow-up of cases**

	N	%
Pregnant women with unknown follow-up	42	
Miscarriages/stillbirths	3/42	7%
Left country before birth	4/42	10%
Newborns to follow-up	35/42	83%
Recovered newborns	31/35	89%
Infants controlled at 9m-old by paediatricians at hospitals	12/31	39%
Infants needed for foolow-up in Primary Health Centres	19/31	61%
Infants without correct follow-up in Primary Health Centres	14/19	74%
Controlled newborns	29/31	94%
Positive T. cruzi newborns	2/29	6.9%

(Source: Informe annual 2010 - Vigilància epidemiològica del protocol de cribratge I diagnòstic de la malaltia de Chagas en dones embarassades llatinoamericanes i els seus nadons. Agència de Salut Pública de Catalunya. Generalitat de Catalunya, 2010).

## Discussion

ASPCAT initiated diagnosis of congenital CD in 2010 with the aim of controlling all pregnant women originating from Latin America who had attended the sexual and reproductive units in Catalonia. Spain. The cost-effectiveness of screening for CD in pregnant women from Latin America in non-endemic areas has already been demonstrated [17]. All pregnant women in Catalonia have a health handbook in which midwives and obstetricians register all the controls that have been carried out during visits. According to the Protocol, the handbook provides a new space to register whether Latin American pregnant woman have been screened for CD during pregnancy. After a few months of application of the Protocol, midwives were aware of CD because most of the eligible women had registered their CD status in the gestation handbook as collected as a representative sample in different primary health centres.

Despite such effective control during pregnancy, ASPCAT was unable to determine the final CD status for 20% of the newborns in 2010. Of importance from a paediatric point of view, most of the CD-infected newborns were asymptomatic, generating a false perception that the newborn was not infected with *T. cruzi*[18]. Follow up of these newborns is important, however, because it has been shown that prompt treatment during the first 12 months of life cures all of them for a lifetime [19, 20].

It has been demonstrated also that screening for CD in pregnant women or newborns provides an opportunity to detect further cases among siblings and relatives as infections often occur in clusters [21]. Only a few cities or regions in Western Europe have implemented screening programmes for congenital CD; Catalonia was one of the first to apply a Protocol for this issue [14]. Contact between hospitals, ASSIRs and primary health care paediatricians, however, must be improved. One of the likely causes of the need for such active surveillance is the lack of good networking between different health providers in this setting, which is insufficient and needs to be strengthened.

The poor control of newborns from CD-positive mothers in paediatric primary health care centres highlights the need for training in congenital CD addressed to those paediatricians. Equally important are actions to improve coordination among different health actors. Community health actions as “expert patient woman” addressed to patients would be very useful to sensitize Latin American community about the importance of control in children to detect congenital CD transmission. Experience to date suggests that the most effective strategies are those that promote “face-to-face” actions among influential groups organized into networks of community action.

This model could be key in strategies proposed by epidemiological surveillance programmes and may be transferable to other areas [22, 23]. This is a long-term investment that benefits the entire community. Finally, but no less importantly, are the sociocultural aspects of CD [24, 25], which should be taken into account in order to improve knowledge of the feelings of CD mothers when they learn that their offspring are at risk for *T. cruzi* infection. It should be fundamental that pregnant CD-infected women understand the need for their children to be followed-up during the first year of life to rule out the possibility of congenital *T. cruzi* infection to them.

## Conclusions

It is essential to implement active surveillance, education and information activities at paediatric primary care and community levels to avoid the loss of CD-infected mothers and their newborns. Training sessions addressed to paediatricians and other involved health professionals would consolidate surveillance and care reference circuits and improve the control of congenital CD.

## References

[1] Prata A (2009). Clinical and epidemiological aspects of Chagas disease. Lancet Infect Dis.

[2] Guhl F, Lazdins-Helds JK, PAHO (2005). Reporte Sobre la Enfermedad de Chagas. Reporte del Grupo de Trabajo Científico Sobre la Enfermedad de Chagas 17-20/04/2005.

[3] World Health Organization (2010). Working to Overcome the Global Impact of Neglected Tropical Diseases: First WHO Report on Neglected Tropical Diseases.

[4] World Health Organization (2013). Sustaining the Drive to Overcome the Global Impact of Neglected Tropical Diseases: Second WHO Report on Neglected Tropical Diseases.

[5] Schofield CJ, Jannin J, Salvatella R (2006). The future of Chagas disease control. Trends Parasitol.

[6] Bern C, Montgomery SP, Herwaldt BL, Rassi A, Marin-Neto JA, Dantas RO, Maguire JH, Acquatella H, Morillo C, Kirchhoff LV, Gilman RH, Reyes PA, Salvatella R, Moore AC (2007). Evaluation and treatment of Chagas disease in the United States: a systematic review. JAMA.

[7] Coura JR, Vinas PA (2010). Chagas disease: a new worldwide challenge. Nature.

[8] Ministerio de Sanidad y Consumo –España (2005). Real Decreto 1088/2005 por el que se establecen los requisitos técnicos y condiciones mínimas de la hemodonación y de los centros y servicios de transfusión. Boletín oficial del estado.

[9] Carlier Y, Torrico F, Sosa-Estani S, Russomando G, Luquetti A, Freilij H, Albajar Vinas P (2011). Congenital Chagas disease: recommendations for diagnosis, treatment and control of newborns, siblings and pregnant women. PLoS Negl Trop Dis.

[10] Howard EJ, Xiong X, Carlier Y, Sosa-Estani S, Buekens P (2014). Frequency of the congenital transmission of *Trypanosoma cruzi*: a systematic review and meta-analysis. BJOG.

[11] Riera C, Guarro A, Kassab HE, Jorba JM, Castro M, Angrill R, Gállego M, Fisa R, Martin C, Lobato A, Portús M (2006). Congenital transmission of *Trypanosoma cruzi* in Europe (Spain): a case report. Am J Trop Med Hyg.

[12] Muñoz J, Coll O, Juncosa T, Vergés M, del Pino M, Fumado V, Bosch J, Posada EJ, Hernandez S, Fisa R, Boguña JM, Gállego M, Sanz S, Portús M, Gascón J (2009). Prevalence and vertical transmission of *Trypanosoma cruzi* infection among pregnant Latin American women attending 2 maternity clinics in Barcelona, Spain. Clin Infect Dis.

[13] Public Health Agency of Catalonia (2010). Protocol for Screening and Diagnosing Chagas Disease in Pregnant Latin American and Their Newborns.

[14] Basile L, Oliveira I, Ciruela P, Plasencia A (2011). Working Group for developing the Catalonian screening programme for congenital transmission of Chagas disease. The current screening programme for congenital transmission of Chagas disease in Catalonia, Spain. Euro Surveill.

[15] Public Health Agency of Catalonia (2010). Vigilància Epidemiològica del Protocol de Cribratge i Diagnostic de la Malaltia de Chagas en Dones Embarassades Llatinoamericanes i els Seus Nadons. Informe Anual 2010.

[16] Institut d’estadística de Catalunya (IDESCAT) (2014). Població Estrangera per Municipis, Catalunya 2013 [Internet].

[17] Sicuri E, Muñoz J, Pinazo MJ, Posada E, Sanchez J, Alonso PL, Gascon J (2011). Economic evaluation of Chagas disease screening of pregnant Latin American women and of their infants in a non endemic area. Acta Trop.

[18] Freilij H, Altcheh J (1995). Congenital Chagas’ disease: diagnostic and clinical aspects. Clin Infect Dis.

[19] Schijman AG, Altcheh J, Burgos JM, Biancardi M, Bisio M, Levin MJ, Freilij H (2003). Aetiological treatment of congenital Chagas’ disease diagnosed and monitored by the polymerase chain reaction. J Antimicrob Chemother.

[20] Neto EC, Rubin R, Schulte J, Giugliani R (2004). Newborn screening for congenital infectious diseases. Emerg Infect Dis.

[21] Zulantay I, Apt W, Ramos D, Godoy L, Valencia C, Molina M, Sepúlveda E, Thieme P, Martínez G, Corral G (2013). The epidemiological relevance of family study in Chagas disease. PLoS Negl Trop Dis.

[22] Singh P, Choksh DA (2013). Community health workers – a local solution to a global problem. N Engl J Med.

[23] Ospina JE, Orcau A, Millet JP, Sánchez F, Casals M, Caylà JA (2012). Community health workers improve contract tracing among immigrants with tuberculosis in Barcelona. BMC Public Health.

[24] Ventura-Garcia L, Roura M, Pell C, Posada E, Gascón J, Aldasoro E, Muñoz J, Pool R (2013). Socio-cultural aspects of Chagas disease: a systematic review of qualitative research. PLoS Negl Trop Dis.

[25] Briceño-León R, Galván JM (2007). The social determinants of Chagas disease and the transformations of Latin America. Mem Inst Oswaldo Cruz.

[CR26] The pre-publication history for this paper can be accessed here: http://www.biomedcentral.com/1471-2458/14/1201/prepub

